# The Impact of Light Availability on the Functional Traits of *Quercus Robur* L. and *Acer Platanoides* L. First-Year Seedlings by Direct and Indirect Methods

**DOI:** 10.1093/icb/icaf003

**Published:** 2025-01-30

**Authors:** Olena Blinkova, Roma Żytkowiak, Andrzej M Jagodziński

**Affiliations:** Institute of Dendrology, Polish Academy of Sciences, Parkowa 5, 62-035 Kórnik, Poland; Department of Horticulture and Ecology, Taras Shevchenko Lugansk National University, 3 Koval St., 36003 Poltava, Ukraine; Institute of Dendrology, Polish Academy of Sciences, Parkowa 5, 62-035 Kórnik, Poland; Institute of Dendrology, Polish Academy of Sciences, Parkowa 5, 62-035 Kórnik, Poland

## Abstract

The resource strategy of seedlings is an important aspect for understanding the adaptation of trees at this ontogenetic phase to abiotic changes. In this study, we sought to determine the patterns of response of functional traits of a shade-tolerant (*Acer platanoides*) and a shade-intolerant (*Quercus robur*) species along natural environmental light gradients. We conducted trait-based analyses at both individual and community levels using direct (leaf area index [LAI] and diffuse noninterceptance [DIFN]) and indirect (light coefficient, derived from Ellenberg values [LC]) methods in the Arboretum at Kórnik (Poland). Differences between the two species were found for some variables. Analysis of phenotypic plasticity indices of leaf, stem, and root traits of seedlings had high values ​​for both species. The values of plasticity indices of *A. platanoides* root traits were lower compared to the corresponding traits for *Q. robur*. Relationships between measures obtained from individual-level trait data were stronger than relationships with measures obtained from community-level trait data. The data obtained from the direct method, which included light measurements both at the community level (experimental plots) and at the individual level (seedlings), revealed the closest relationships between functional traits of seedlings and light changes at the individual level trait data for both species. Correlation links between LAI and leaf (leaf mass per area; specific leaf area) and stem (specific stem length; stem mass fraction) traits were less tight for *Q. robur* compared to *A. platanoides*. The indirect Ellenberg indicator analysis revealed relationships between LC and leaf mass per area, and stem-to-root ratio of seedlings based on community-level trait data. Close relationships between LC and leaf mass fraction and specific leaf area were not established, in contrast to LAI and DIFN. The closest relationships, representing among traits within the same organ system, and links, describing interactions between traits of different organ systems, were established at the community-level trait data.

## Introduction

The link between functional characteristics of plants and their habitats has always been an important component of plant science (Grime 1974; Violle et al. 2007; de la Riva et al. 2016a, 2016b; Bruelheide et al. 2018; Yin et al. 2018; Kattge et al. 2020). Plant functional trait-based research spanning multiple levels of biological organization, from cells and tissues to individual organisms and populations to communities and ecosystems, is an important tool for studying many ecological issues (Lavorel and Garnier 2002; Valladares and Niinemets 2008; Fortunel et al. 2012; Laughlin and Laughlin 2013; Roscher et al. 2013; Valladares et al. 2015; Diaz et al. 2016). It is common knowledge that plant economics spectrum theory attempts to integrate leaf, stem, and root traits to explain plant ecological strategies (Walters and Reich 1996; Reich et al. 1998; Wright et al. 2004; Diaz et al. 2016; Kattge et al. 2020). The theory predicts that the above-ground and below-ground traits, such as specific leaf area (SLA), leaf mass per area (LMA), and specific root length (SRL), are correlated with each other and coordinated across different organs, and functional traits should have adaptive significance along environmental gradients in temperate broadleaf forests (de la Riva et al. 2016a), subarctic flora (Freschet et al. 2010) and Mediterranean rangeland (Perez-Ramos et al. 2012). The leaf, stem, and root traits should respond to environmental gradients, with light primarily influencing above-ground traits and indirectly shaping root traits through its impact on carbon allocation and resource trade-offs (Poorter et al. 2012; Reich 2014; Kramer-Walter et al. 2016; Valverde-Barrantes et al. 2017). Economic traits of plants influence performance and fitness consistent with trait-based theory about underlying adaptive mechanisms (Reich 2014). However, some studies did not support consistent coordination between above-ground and below-ground traits. Root traits mirrored stem traits rather than leaf traits in a Neotropical region (Fortunel et al. 2012). The leaf and stem economic spectra were not only independent (Wright et al. 2004), but also orthogonal (Baraloto et al. 2010), suggesting that functional trade-offs operate independently at the leaf and stem levels (Wright et al. 2007). Many root, stem, and leaf traits of plants were coordinated, but at the species level, root traits were not clearly related to growth rate and loaded on a separate principal component from the plant economic spectrum (Kramer-Walter et al. 2016). Some studies show that roots are subject to a more complex environment than above-ground traits (Shen et al. 2019). Above-ground traits cannot adequately capture the variety of below-ground functions and trade-offs that drive differences in plant performance across species (Kramer-Walter et al. 2016; Valverde-Barrantes and Blackwood 2016; Valverde-Barrantes et al. 2017). The relationships between leaf and root traits can shift depending on soil properties (Hu et al. 2019), water availability (Fort et al. 2017), and other abiotic factors (Valverde-Barrantes et al. 2017). Consequently, some functional plant trait variations might be uncorrelated due to ecological variabilities in above- and below-ground environments (Qi et al. 2020).

The study of the morphological/ecological characteristics and functional traits of tree seedlings is of keen interest among recent ecological submissions. The seedling stage of trees is particularly critical because survival and performance at this ontogenetic phase of tree development will characterize the future of forest/park ecosystems (Khan et al. 2021). All the same, seedling functional types refer to the morphology of seedlings in relation to cotyledon function and position. It is a categorical trait that can be used to characterize plant ecological adaptations (Perez-Harguindeguy et al. 2013). Seedling survival of trees of different functional types has an imprint on seedling and adult community structure. This ecological aspect requires knowledge regarding how individuals interrelate with habitat. Seedling above-ground and below-ground functional traits (Supplementary Table S1) enable us to quantify strategies for different environments (He et al. 2019, 2020). A number of functional traits contribute to seedling persistence in the forest understory (Kitajima 1994; Valladares et al. 2002; Kitajima and Poorter 2008). Most seedling traits depend on the environments, either marginally changing (weak trait × environment interaction) or reversing (strong trait × environment interaction) along abiotic gradients (Li et al. 2022). At this stage, seedlings rely heavily on resources stored in the seed and their functional traits (e.g., SLA, LMA, and root traits) and are highly responsive to environmental gradients, such as light availability (Kitajima 1994; Poorter et al. 2012; Perez-Harguindeguy et al. 2013; O’Donnell et al. 2020).

Intensity of light is a key parameter for seedling development, and thus it is capable of influencing forest composition, structure, and dynamics (Lusk 2004; Lusk and Piper 2007). Survival of seedlings is closely correlated with seed mass in low light, whereas the relationship becomes insignificant in intensive light (Poorter and Rose 2005). At low irradiance, species deviate most in their morphological characteristics because light interception is crucial in a light-limited environment. At high irradiance, species deviate most in their physiological characteristics because high irradiance allows the species to realize their full photosynthetic capacity and growth potential (Kitajima 1994; Veneklaas and Poorter 1998; Poorter 1999; Poorter et al. 2012; Perez-Harguindeguy et al. 2013). Several studies have documented the large variation displayed by functional traits under different light regimes, along with their effects on survival and plant growth. Traits such as SLA and LMA are widely recognized as critical indicators of light capture efficiency and structural investment, respectively (Kitajima 1994; Veneklaas and Poorter 1998; Niinemets and Valladares 2006). Shade-tolerant species typically exhibit higher SLA values, enabling effective light interception under low-light conditions, whereas shade-intolerant species prioritize traits such as higher LMA for durability and growth optimization under high irradiance (Kitajima 1994; Niinemets and Valladares 2006; Poorter et al. 2012).

While SLA and LMA are critical for light capture and photosynthetic efficiency, below-ground traits such as SRL and root mass fraction (RMF) reflect the plant’s ability to acquire resources in the soil, highlighting the complementary roles of above-ground and below-ground adaptations. Shade-tolerant species tend to allocate a higher proportion of biomass to roots (higher RMF) to enhance nutrient uptake in shaded environments, while shade-intolerant species allocate more to above-ground growth to capitalize on light availability (Valladares et al. 2002; Kramer-Walter et al. 2016).

Understanding the coordination of functional traits at different levels is essential for assessing plant adaptation strategies. Parameters that describe the relationships among traits within the same organ system (referred to as “within-organ system” correlations), such as SLA and LMA within leaves, and parameters that represent the interactions between traits of different organ systems (referred to as “between-organ system” correlations), such as the relationships between leaf traits and root or stem traits. These coordinated relationships reflect both the intra-organ integration of functions and inter-organ responses to environmental gradients, such as light availability (Freschet et al. 2015).

Low light availability in the understory of natural communities primarily results from overlying plant canopies and the potential occurrence of differently sized gaps (Canham et al. 1994; Valladares et al. 2006). These environmental gradients influence seedling traits and survival strategies across species. Shade-tolerant species exhibit adaptations that allow them to persist under low-light conditions, often at the expense of rapid growth, while shade-intolerant species display higher growth rates in high-light environments but lower survival in the shade (Kitajima 1994; Poorter and Rose 2005). Seedlings of shade-tolerant and shade-intolerant species, such as *Acer platanoides* and *Quercus robur* in this study, exhibit differences in survival and growth strategies under varying light conditions. These patterns align with general findings in prior studies across a range of species (Canham et al. 1994; Kitajima 1994; Valladares et al. 2006).

However, only some of the shade-tolerant species have low rates of sapling mortality under the low light levels characteristic of stands dominated by late-successional species (Canham et al. 1994). It is also important to understand the light requirements of coexisting species during different stages of development. This trait varies among coexisting species, showing a continuum of seedling performance strategies (Kobe et al. 1995; Valladares et al. 2015) and the position along the growth-survival trade-off (Capers et al. 2005). Growth rates for different light survivorship strategies are inversely correlated across coexisting species (Kobe et al. 1995). Seedlings of shade-intolerant species occurred at significantly higher light levels than the shade-tolerant species. The proportion of seedlings in low-light conditions was negatively correlated with the successional position of the species (Poorter and Arets 2003). The differences in light use between strategies are invoked to explain seedling structure and performance through optimum growth and survival relationships. The species face a trade-off between a high survival rate in the deep shade and a high growth rate in high light conditions (Kitajima 1994).

Shade-tolerant and shade-intolerant species therefore differ in a predictable way in their seedling traits, such as SLA, LMA, RMF, and SRL, which reflect their respective strategies for optimizing growth and survival under contrasting light conditions. These traits not only reflect their ecological strategies but also highlight the role of phenotypic plasticity in enabling species to adapt to variable environments. Phenotypic plasticity, defined as the ability of a single genotype to express different phenotypes in response to environmental conditions, is critical for survival in heterogeneous light environments (Valladares et al. 2006). For instance, shade-tolerant species often exhibit high plasticity in leaf traits, allowing them to maximize light capture in shaded conditions, while shade-intolerant species display plasticity that supports rapid growth in high-light environments. A new approach demonstrated that the plasticity in some plant traits may represent specialization (Lortie and Aarssen 1996). The plastic response for morphological parameters to environmental deterioration is adaptive only if the alternatives are dormancy or death and is generally less adaptive than phenotypic stability (Valladares et al. 2006). The study of phenotypic plasticity of morphological parameters of leaves, stems, and roots of shade-tolerant and shade-intolerant seedlings is especially relevant in changing environmental conditions. The plant ecological spectrum/phenotypic plasticity and seedling traits are coordinated, but to what extent does the strength of this link vary with the different light availabilities? Shade-tolerant species grow slowly and have higher survival rates under low light conditions characteristic of the forest understory, whereas shade-intolerant species grow quickly but have lower survival rates in shaded environments, thriving instead in high light conditions such as open gaps (Han et al. 2017). The functional traits should be predictors of demographic rates, but results in the literature are mixed, indicating that additional information regarding organismal function should be considered. Most of the scientific research literature on this topic was carried out in natural tropical and temperate forests. However, information regarding conducting research under ex situ conditions is not sufficiently demonstrated, despite its critical importance for isolating species-specific traits and phenotypic plasticity, which are essential for understanding the reproduction and conservation of plant biodiversity.

*Acer platanoides* L. (a shade-tolerant species) is the most widespread native maple in Europe and is generally found in small groups or as scattered individuals in mixed forests (Nowak and Rowntree 1990). *Quercus robur* L. (a shade-intolerant species) is also among the most important tree species in Central Europe, both from ecological and economical points of view (Jones 1959). *Acer platanoides* and *Q. robur* are frequently noted seedlings in forests in Poland (Adamczak 2006). Besides, the maple (Lapointe and Brisson 2012) and oak (O’Donnell et al. 2020) seedlings can grow under a canopy of other trees. The plant economics spectrum shows that ecological traits of seedlings of both species, such as SLA, LMA, LAR, RMF, and SRL, are functionally coordinated along a light gradient, reflecting their integrated strategies for adaptation to varying light availability. However, empirical evidence is mixed about whether above-ground and below-ground traits are consistently linked. We expect links of light parameters and a number of functional traits of the established seedling phase of *A. platanoides* and *Q. robur*. The community-weighted mean (CWM) allows us to assess how the functional traits of seedlings collectively respond to environmental gradients, providing insights into community-level ecological strategies. This approach is particularly important for understanding how shared growth conditions, such as light availability and interspecific interactions, shape the combined adaptation strategies of *A. platanoides* and *Q. robur* seedlings.

We predict that: (1) changing light availability (direct and indirect methods) and functional parameters of seedlings (leaves, stems, roots) of model shade-tolerant (*A. platanoides*) and shade-intolerant (*Q. robur*) species at the individual and CWM levels are correlated; (2) variation exists in the quantified phenotypic plasticity (plasticity index [PI]) of functional traits of seedlings and local environments; (3) parameters of the functional characteristics of shade-tolerant (*A. platanoides*) and shade-intolerant (*Q. robur*) seedlings are correlated between traits of different organ systems and traits within the same organ system. The current study aimed to investigate functional traits of first-year seedlings of two key tree species of Poland in behavioral response to light variability.

## Materials and methods

### Study site and experimental design

**Fig. 1:**
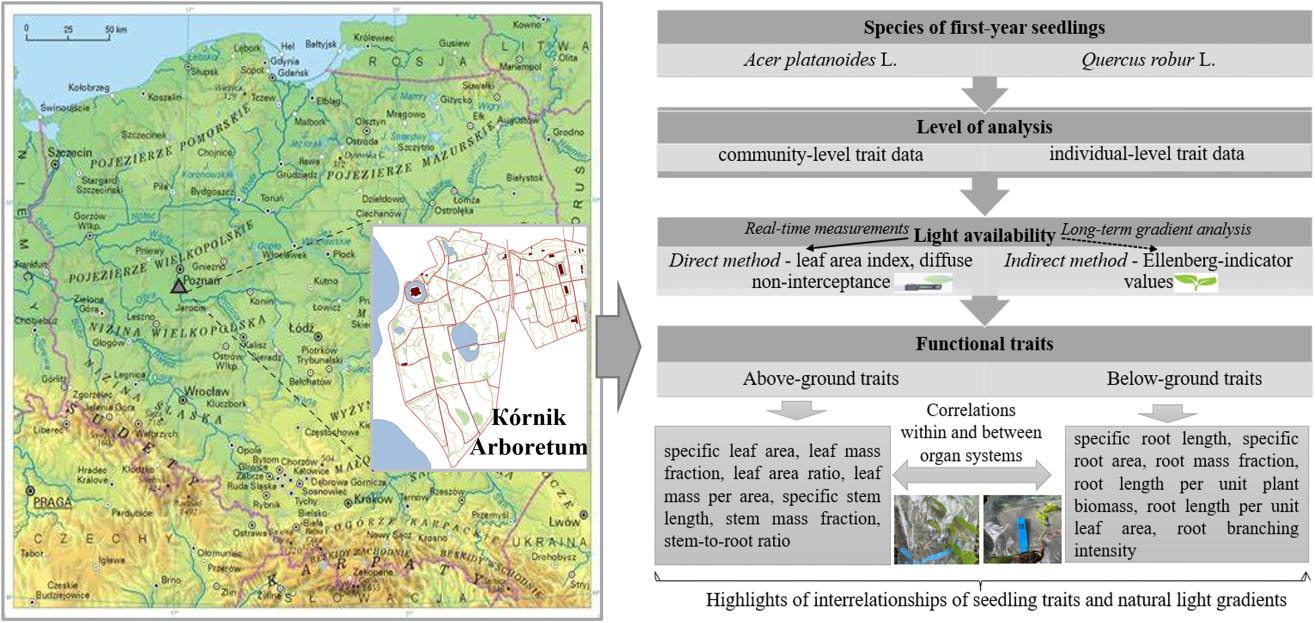
Schematic overview of the study design.

The research was conducted during May–June 2023 on the territory of the Kórnik Arboretum (Western Poland; 52.2448°N, 17.09698°E, 75 m a.s.l.); from 10:00 to 12:00 AM daily; ten 50 × 50 m experimental plots (EP l–10) were laid out. Five randomly located 1 × 1 m subplots were arranged within each EP (1–10), the total number of *Q. robur* and *A. platanoides* seedlings was tallied, and the distance between individuals was measured. All EPs were established within the Kórnik Arboretum in areas with naturally varying light availability due to differences in canopy cover. The overall experimental design and methodology are shown in Fig. 1 to provide a visual overview of the study framework.

Light conditions were not experimentally manipulated but were measured as they naturally occurred, providing an ecologically relevant gradient for studying seedling functional traits. Seedlings are defined as first-year individuals of tree species (*A. platanoides* and *Q. robur*) that have germinated during the most recent growing season. These seedlings are characterized by the presence of cotyledons or residual cotyledon scars and the absence of secondary woody growth, ensuring that they represent the early ontogenetic stage of development. The seedling category of the species was based on three cotyledon characters of presumed ecological significance: position (epigeal or hypogeal), texture (fleshy or papery), and exposure (exposed or protected), as established by Garwood (1996). Including this level of detail highlights the relevance of these traits in understanding seedling survival and growth under varying environmental conditions. On each subplot, three seedlings of each of the two species were randomly selected and excavated for measuring leaf, stem, root, and entire-plant traits (combination of above-ground and below-ground functional traits, Supplementary Table S1).

### Indirect method: Ellenberg indicator analysis

To collect the geobotanical data, five subplots of 5 × 5 m (25 m^2^) were arranged within each EP (1–10). In each EP, five subplots were located north, east, south, west, and around a central point of the EP. These subplots were not associated with 1 × 1 m subplots (2.1). Herb species identification to species level occurred in the field and was verified in the laboratory. We used Ellenberg indicator values (Ellenberg 1998; Didukh 2012; Tichy et al. 2023) to assess environmental conditions indirectly based on the herbaceous species composition in each plot. Ecological optima represent the environmental conditions under which plant species perform best in terms of growth and survival. Using Ellenberg indicator values (Ellenberg 1998; Didukh 2012; Tichy et al. 2023), we identified the environmental optima of herbaceous species in each plot for light (LC). Each species was assigned an ecological indicator value for light based on established ecological databases (Ellenberg 1998; Didukh 2012; Tichy et al. 2023). These values were used to infer light gradients across the plots, providing a comprehensive characterization of site conditions. This indirect method allows comparison of the obtained results with direct measurements of light availability and provides a more detailed understanding of how environmental factors influence seedling traits. The environmental indicators of soil properties were identified using unified scales of environmental amplitudes, which assign species-specific indicator values for factors such as light (LC) based on ecological optima and tolerance ranges (Ellenberg 1998; Ellenberg and Leuschner 2010; Holtland et al. 2010; Didukh 2012; Tichy et al. 2023). LC is an important factor affecting the coenosis composition (Ellenberg 1998; Ellenberg and Leuschner 2010). LC takes values from 1 on heavily shaded soils to 9 on soils receiving full sunlight. The mean of indicator value was calculated as weighted averages based on the presence/absence of species on EPs.

### Direct method: light measurements

Measurements of canopy structure and light availability were taken using the LAI-2200 plant canopy analyzer (Li-Cor Inc., Lincoln, NE, USA), which records leaf area index (LAI, the area of leaves per unit ground area, m²/m², representing foliage density) and diffuse noninterceptance (DIFN, the fraction of diffuse light passing through the canopy, reflecting canopy openness and light availability at the understory level). Observed values of LAI and DIFN in our study represent varying levels of canopy openness in natural temperate forest conditions. These values are consistent with typical measurements of light availability reported in temperate forest ecosystems (Tinya et al. 2009; Dąbrowski et al. 2013). For each experimental plot (EP 1–10), we took five series of twenty measurements at the height of 0.5 m above ground (Machado and Reich 1999). These five series were associated with collected geobotanical subplots (2.2). The measurements were collected during bright (clear) sky conditions. Light availability measurement was also recorded at a height of 10 cm above individual seedlings of *Q. robur* and *A. platanoides* on all subplots (2.1). For each individual seedling, we took four measurements at a time.

The combination of Ellenberg indicator analysis (indirect method) and direct light measurements (LAI, DIFN) enables complementary perspectives on light availability. The indirect method captures long-term environmental gradients based on plant community composition, while the direct method provides precise, real-time data on canopy structure and diffuse light penetration. Together, these approaches provide a comprehensive understanding of how light availability influences seedling functional traits under ecologically relevant conditions.

### Seedling above- and below-ground biomass

Tree seedlings were carefully rinsed and individually separated into fractions: leaves (with petioles), stems, and roots. All the samples were oven-dried at 65°C (ULE 600 and UF450, Memmert GmbH + Co. KG, Germany) and weighed using BP 210 S (Sartorius, Göttingen, Germany) and Mettler Toledo PG 1003-S (Mettler Toledo, Columbus, OH, USA) scales (±0.001 g). A hand shovel was used to excavate the root systems, carefully sampling all the roots. Total seedling biomass (BIOM), stem biomass, leaf biomass, and root biomass were measured. The trait mass fractions (Supplementary Table S1) were calculated as (relative) dry mass per individual.

### Leaf morphological characteristics

The leaf samples for morphological traits of tree seedlings were collected on the same day. The leaves per individual were harvested for both species, put in plastic bags, and transported to the lab. In the lab, all the samples were imaged and scanned using WinFOLIA 2013 PRO software (Regent Instruments Inc., Quebec, Canada). Based on the measurements, we calculated leaf mass per area (LMA). We further calculated the leaf mass fractions (LMFs), specific leaf area (SLA), and leaf area ratio (LAR) (Supplementary Table S1). The leaf traits were calculated per individual plant (Walters and Reich 1996; Gomez-Aparicio et al. 2008).

### Stem morphological characteristics

The stem samples for morphological traits of tree seedlings were collected on the same day too. The stems per individual were harvested for both species, put in plastic bags, and transported to the lab. The length of stem was measured. The specific stem length (SSL), stem mass fraction (SMR), and stem-to-root ratio (S/R) were calculated (Supplementary Table S1).

### Root morphological characteristics

The root samples of tree seedlings were thoroughly shaken to remove soil particles and placed in plastic bags. In the laboratory, root samples were soaked in water and carefully cleaned. Root morphological parameters, including the mean diameter, total surface area, root length, and volume, were determined per plant. Roots of each order were scanned with a light-transmitting desktop scanner in gray scale at 300 dpi. We used WinRHIZO software (Regent Instruments, Inc.). Specific root length (SRL), specific root area (SRA), root mass fraction (RMF), root length per unit plant biomass (RLPM), and root length per unit leaf area (RLLA) were calculated (Kong et al. 2014). Branching intensity of root (RBI) was calculated too (Liese et al. 2017) (Supplementary Table S1).

### Plasticity index

We quantified phenotypic plasticity using the PI to measure the relative variation in functional traits. An index of phenotypic plasticity, ranging from 0 to 1, was calculated for each functional trait and for the two tree species. The index was derived as the difference between the minimum and maximum mean trait values across natural light gradients, divided by the maximum mean value (Valladares et al. 2000). Higher values of the index indicate greater phenotypic flexibility in response to environmental changes, while lower values reflect greater stability or consistency in trait expression across varying conditions. This method emphasizes species-level plasticity, providing a standardized and widely accepted approach for comparing central tendencies of plastic responses across natural light gradients. The chosen approach for assessing phenotypic plasticity is standard for field conditions, where data are collected once for each plant. We acknowledge that the Relative Distance Plasticity Index (RDPI) method (Valladares et al. 2006) provides a more detailed assessment of plasticity at the individual level, but it could not be applied in our study due to the absence of repeated measurements for the same plant. While this calculation does not capture within-species variability, it allows for robust interspecific comparisons, offering valuable insights into how species respond to environmental gradients under varying natural light conditions.

### Community-weighted mean

The CWM of each functional trait was calculated for each 1 × 1 m subplot as the sum of the product of the trait values (above-ground and below-ground traits) of both species by its relative abundance (Garnier et al. 2004).


\begin{eqnarray*}
{\bf{CWM}} = \mathop \sum \nolimits_{t = 1}^n {P_i} \times \mathit{trai}{t_t},
\end{eqnarray*}

where *n* is the species number of a seedling plot; *P_i_* is the relative abundance of species (total abundance in each seedling plot was used), and *trait_i_* is the trait value of each species.

### Statistical analysis

Summary statistics were calculated, including mean, median, min, max, standard deviation, standard error, and variability (coefficient of variation) for *A. platanoides* and *Q. robur* seedling traits (BIOM, LA, LMA, LMF, LAR, SLA, SSL, SMR, S/R, RMF, SRA, SRL, RLPM, RLLA, RBI; Table 1). Linear regression analysis was used to predict the value of LAI on the values of leaf, stem, and root functional traits at the individual level. Linear regression analysis was used to predict the values of LAI, DIFN, and LC on the value of CWM leaf, stem, and root traits. We used a plot correlation matrix (Pearson, *P* < 0.05, *P* < 0.01, *P* < 0.001) to examine the correlations among traits within the same organ system and correlations between traits of different organ systems, focusing on the relationships between functional traits: leaf, stem, and root. We conducted a principal component analysis (PCA) to relate the variations of indices of functional traits to the determined light factors. For the analytical processing of the field and laboratory data, the calculation was performed using OriginPro 2022 software (Version 9.9.0.225, OriginLab Corporation, Northampton, MA, USA).

**Table 1:** *Acer platanoides* and *Quercus robur* seedling descriptive parameters

Trait	Mean		Median		Min		Max		σ		CV, %	
	*A. platanoides*	*Q. robur*	*A. platanoides*	*Q. robur*	*A. platanoides*	*Q. robur*	*A. platanoides*	*Q. robur*	*A. platanoides*	*Q. robur*	*A. platanoides*	*Q. robur*
BIOM	0.08	1.13	0.07	1.01	0.01	0.17	0.35	3.60	0.05	0.59	62.51	52.21
LA	15.34	37.29	14.07	33.70	3.51	10.02	34.02	79.80	6.97	19.95	45.43	53.49
LMA	2.22 × 10^−3^	2.92 × 10^−3^	2.21 × 10^−3^	2.87 × 10^−3^	1.14 × 10^−3^	1.89 × 10^−3^	4.22 × 10^−3^	4.82 × 10^−3^	6.66 × 10^−4^	5.44 × 10^−4^	26.11	18.63
LMF	0.41	0.33	0.43	0.35	0.06	0.01	0.58	0.69	0.11	0.10	26.82	30.33
LAR	192.78	62.83	185.18	36.53	13.65	3.54	185.18	156.52	57.54	28.87	29.85	47.50
SLA	485.92	355.79	450.51	349.00	237.00	239.00	880.00	528.00	150.75	62.91	31.02	17.68
SSL	429.19	165.58	372.89	158.24	171.58	51.77	1192.02	335.69	216.41	60.53	50.44	36.55
SMR	0.24	0.19	0.23	0.01	0.06	2.78 × 10^−4^	0.37	0.34	0.07	0.07	29.16	36.84
S/R	4.08	0.53	3.29	0.57	1.21	0.07	12.13	1.46	2.24	0.37	54.90	69.81
RMF	0.23	0.28	0.24	0.21	0.08	0.03	0.45	0.77	0.08	0.09	34.78	32.14
SRA	238.11	71.83	226.21	70.85	95.36	36.03	463.25	134.21	73.25	21.80	30.76	30.34
SRL	1311.31	271.84	1213.86	235.85	435.84	84.32	2535.84	567.50	458.55	138.57	34.96	50.97
RLPM	291.63	80.58	276.27	54.91	66.29	79.81	649.09	276.18	128.57	44.36	44.08	55.05
RLLA	1.57	2.12	1.34	1.91	0.50	0.23	3.89	7.69	0.79	1.43	50.31	67.45
RBI	4.23	4.37	4.05	3.83	1.10	2.27	10.05	15.21	2.08	1.93	49.12	44.16

## Results

**Fig. 2:**
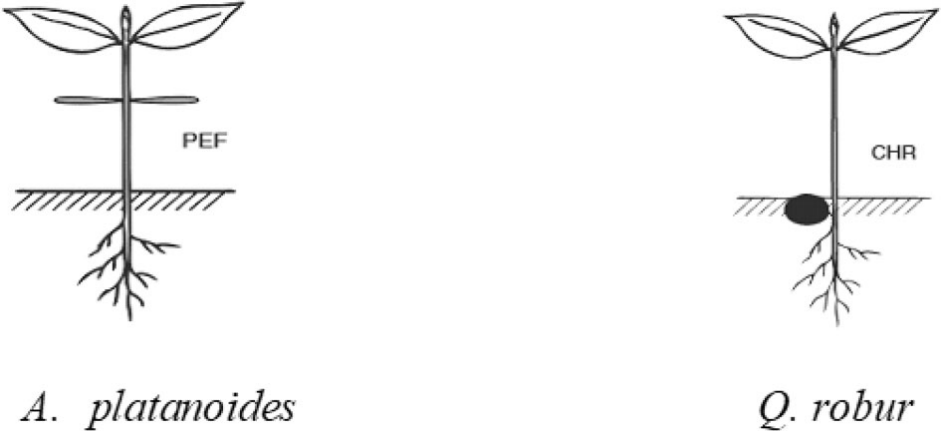
Studied seedlings functional types: (A) *Acer platanoides* (PEF: phanerocotylar epigeal with foliaceous cotyledons); (B) *Quercus robur* (CHR: cryptocotylar hypogeal with reserve storage cotyledons).

Diagnostic and dominant species in the herbaceous layer on the EPs in the Arboretum were *Bromus sterilis* (L.) Nevski, *Convallaria majalis* L., *Impatiens parviflora* DC., *Elytrigia repens* (L.), *Geum urbanum* L., *Geranium robertianum* L., *Melica uniflora* Retz., *Galeobdolon luteum* Huds., and *Galium odoratum* (L.) Scop. The mean foliage cover on EPs was 75–85%. The main canopy tree species were *A. platanoides, Q. robur, Carpinus betulus* L., and *Abies alba* Mill. Development of first-year seedlings only of *A. platanoides* and *Q. robur* was detected on the EPs. The functional types established were phanerocotylar epigeal with foliaceous cotyledons (PEF) for *A. platanoides* (Fig. 2a) and cryptocotylar hypogeal with reserve storage cotyledons (CHR) for *Q. robur* (Fig. 2b). The cotyledon position and function, reflecting the adaptive strategies of each species during early development, are shown in Fig. 2.

### Comparative analysis of leaf, stem, and root traits/plasticity indices of tree seedlings

**Fig. 3:**
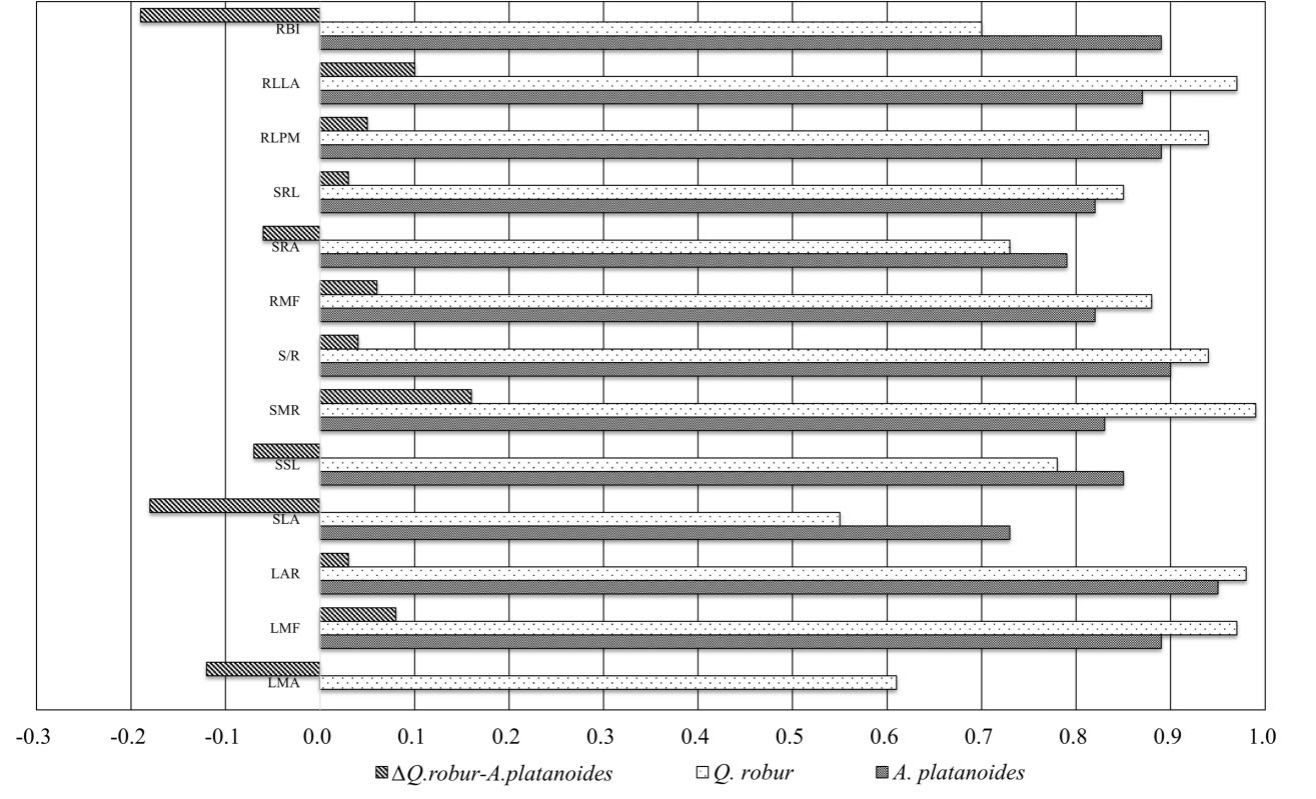
Plasticity indices of functional traits of *Quercus robur* and *Acer platanoides*. Above-ground traits—SLA: specific leaf area, LMF: leaf mass fraction, LAR: leaf area ratio, LMA: leaf mass per area, SSL: specific stem length, SMR: stem mass fraction, S/R: stem-to-root ratio; and below-ground traits—SRL: specific root length, SRA: specific root area, RMF: root mass fraction, RLPM: root length per unit plant biomass, RLLA: root length per unit leaf area, RBI: root branching intensity. This figure highlights the contrasting adaptive strategies of shade-tolerant (*A. platanoides*) and shade-intolerant (*Q. robur*) species.

The calculated values ​​of functional traits of *A. platanoides* and *Q. robur* seedlings on the EPs differed in ways that reflected the established functional types of the seedlings (Table 1). The obtained mean/median values ​​of the studied traits corresponded to the ontogenetic stage of development of seedlings and species for forests of the temperate zone. The coefficient of variation of BIOM was higher for *A. platanoides* (62.51%) than for *Q. robur* (52.21%). LA (45.43%) and LAR (29.85%) had the maximum variability among the leaf functional traits of *A. platanoides*. LMA and LMF of *A. platanoides* had almost the same variability. LA and LAR also had (by analogy with *A. platanoides*) the maximum values of variation coefficient among leaf functional traits of *Q. robur*. However, the variation coefficient of LMA was only 18.63% for *Q. robur* seedlings. It is worth noting the different variability of SLA for the studied seedlings: almost twice higher for *A. platanoides* than for *Q. robur*. High variabilities of stem traits (29.16–69.81%) and root traits (30.34–67.45%) were found for seedlings of both species. There was no significant difference in the variability of stem traits for seedlings of *A. platanoides* and *Q. robur*. The maximum value of this parameter was detected for RLLA among root traits: 50.31% (*A. platanoides*) and 67.45% (*Q. robur*). Values of RMF and SRA ​​were similar for both species. The variability of RLPM was slightly higher for *Q. robur* than for *A. platanoides*. PIs of the studied parameters of leaves, stems, and roots were high for seedlings of both species (Fig. 3). The maximum values of ecological plasticity among leaf traits were found for LAR and LMF. Moreover, the SLA PI for *Q. robur* was slightly more than 0.5, while the index was 0.73 for *A. platanoides*. This was consistent with our previous data (3.2). Among stem parameters studied, S/R had the highest plasticity for both species. RLLA (*Q. robur*) had the maximum value of plasticity among root functional traits. RBI of *Q. robur* seedlings had the minimum value of plasticity. Generally, the root functional traits of *A. platanoides* seedlings had lower values of PIs compared to the corresponding parameters for *Q. robur*. The dissimilarity (∆) between the studied traits was the largest for SLA (−0.18), RBI (−0.19), and SMR (0.16). The minimum (∆) was recorded for LAR (0.03), S/R (0.04), SRL (0.03), and RLPM (0.05) (Fig. 3).

### The relationships between light availability and functional traits at the individual level by direct methods

**Fig. 4:**
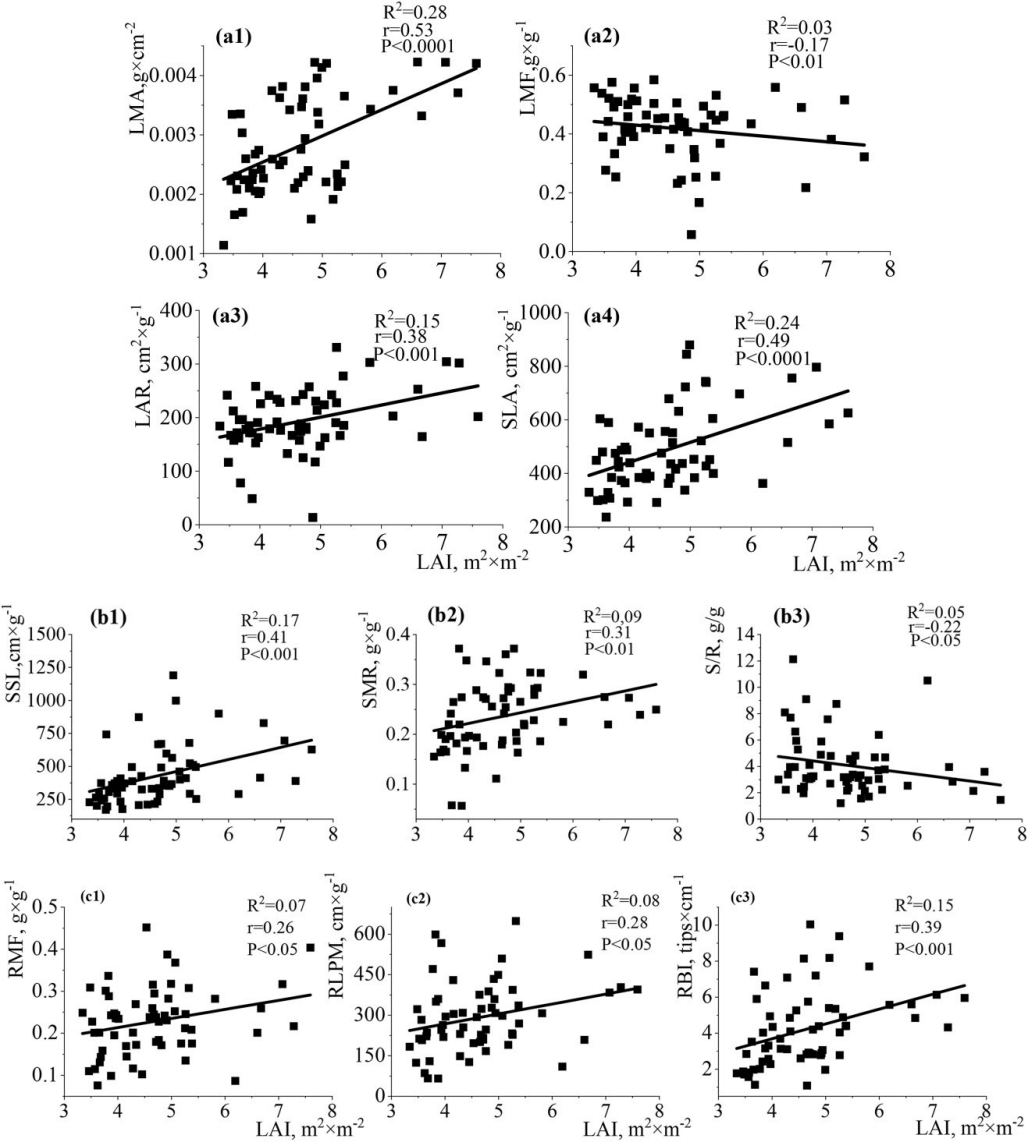
Single-variable linear regression analyses between LAI (leaf area index) and (A) leaf traits (LMA: leaf mass per area—a1; LMF: leaf mass fraction—а2; LAR: leaf area ratio—а3; SLA: specific leaf area—a4), (B) stem traits (SSL: specific stem length—b1; SMR: stem mass ratio—b2; S/R: stem-to-root ratio—b3), and (C) root traits (RMF: root mass fraction—с1; RLPM: root length per unit biomass—с2; RBI: root branching intensity—с3) of *A. platanoides* seedlings. Each point represents individual seedling measurements. This figure highlights the relationships between above-ground and below-ground traits and LAI, emphasizing species-specific responses to light availability.

**Fig. 5:**
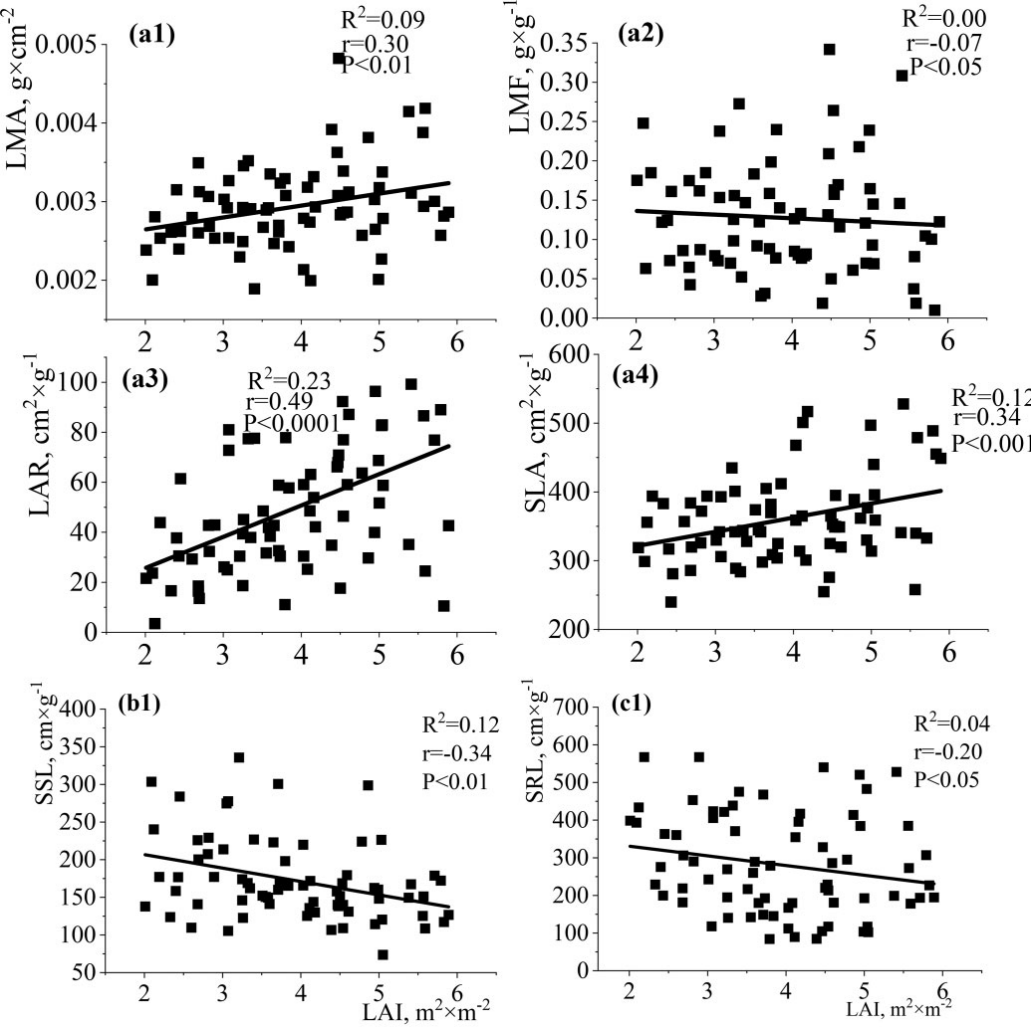
Single-variable linear regression analyses between LAI (leaf area index) and (A) leaf traits (LMA: leaf mass per area—a1; LMF: leaf mass fraction—а2; LAR: leaf area ratio—а3; SLA: specific leaf area—а4), (B) stem trait (SSL: specific stem length—b1), and (C) root trait (SRL: specific root length—c1) of *Q. robur* seedlings. Each point represents individual seedling measurements. This figure highlights the relationships between above-ground and below-ground traits and LAI, emphasizing species-specific responses to light availability.

The relationships between the functional traits of shade-tolerant (*A. platanoides*) and shade-intolerant (*Q. robur*) species (individual level of analysis) and light availability in the studied environmental conditions of the Arboretum showed similar results for *A. platanoides* and *Q. robur* seedlings, but with certain differences (Figs. 4 and 5). The particular contrast of seedlings of both species was between the investigated light conditions and functional traits of stem and root (Fig. B and C; Fig. 5B and C; Supplementary Figs. S1 and S2). The studied morphological leaf traits differed in value per species but were similarly and significantly modified by light levels. For *A. platanoides*, variables SLA/LMA/LAR had significant positive relationships with LAI, with *R*^2^ = 0.24, *r* = 0.49, *P* < 0.0001 for SLA, *R*^2^ = 0.28, *r* = 0.53, *P* < 0.0001 for LMA, and *R*^2^ = 0.15, *r* = 0.38, *P* < 0.0001 for LAR. Conversely, LMF demonstrated a moderate negative relationship with LAI (*R*^2^ = 0.03, *r* = −0.17, *P* < 0.01) (Fig. 4a2). Instead, variables SSL/SMR had positive relationships with LAI with *R*^2^ = 0.17, *r* = 0.41, *P* < 0.01 for SSL and *R*^2^ = 0.09, *r* = 0.31, *P* < 0.01 for SMR, while S/R had a nonsignificant negative trend in the relationships in this study (b1–b3). Leaf and stem traits show correlations with light availability and provide key insights into above-ground adaptation strategies. The obtained results regarding the impact of light availability on the root traits of *A. platanoides* were quite expected (c1–c3, Supplementary Fig. S1). Root traits showed weaker or nonsignificant relationships with light availability, suggesting that they are less indicative of direct light-driven adaptations. The variables RMF/SRA/SRL/RLPM/RLLA had nonsignificant positive relationships with LAI (Supplementary Fig. S1). A moderate relationship was established between LAI and RBI (*R*^2^ = 0.15, *r* = 0.39, *P* < 0.01). The relationships between LAI and SRA/SRL were almost the same strength.

The relationships between the functional traits of *Q. robur* seedlings and the change in light availability revealed trends similar to *A. platanoides* seedlings. However, some differences in relationships were recorded. For *Q. robur*, variables SLA/LMA/LAR had significant positive relationships with LAI (Fig. 5a1, a3, and a4). However, the relationship between LAI and LAR for *Q. robur* was closer (*R*^2^ = 0.23, *r* = 0.49, *P* < 0.0001) compared to *A. platanoides*. The relationships between LAI and LMA (*R*^2^ = 0.09, *r* = 0.30, *P* < 0.01) and SLA (*R*^2^ = 0.12, *r* = 0.34, *P* < 0.01) were weaker for the shade-intolerant (*Q. robur*) species than the shade-tolerant (*A. platanoides*) species under the light conditions studied. The variable SSL had significant negative relationships with LAI (*R*^2^ = 0.12, *r* = −0.34, *P* < 0.01) and SMR, and S/R had nonsignificant positive trends in the relationships in this study (b1, Supplementary Fig. S2). The negative relationship between SSL and LAI (*R*^2^ = 0.12, *r* = −0.34, *P* < 0.01) for *Q. robur* seedlings contrasted with the positive relationship for *A. platanoides* (*R*^2^ = 0.17, *r* = 0.41, *P* < 0.001). The correlation between LAI and SMR was half as large for *Q. robur* as compared to *A. platanoides*. The S/R parameter of *Q. robur* seedlings had the opposite trend compared to *A. platanoides* seedlings (b3, Supplementary Fig. S2). The variables RMF/SRA/SRL/RLPM/RLLA had nonsignificant positive or negative relationships with LAI (Supplementary Fig. S2). The links between the root traits of *Q. robur* seedlings and the studied light availability in the Arboretum were predictable (c1, Supplementary Fig. S2). The relationship between LAI and SRL (*R*^2^ = 0.04, *r* = −0.20, *P* < 0.05) was the most closely related. These root links were substantially weaker than for *A. platanoides*. These results highlight species-specific differences in light-driven trait responses and suggest that at the individual level, *Q. robur* seedlings exhibit lower plasticity in root traits under the studied light conditions compared to *A. platanoides*.

### The relationships between light availability and CWM functional trait values by direct and indirect methods

The analysis at the community-weighted level (absolute abundance of the studied species) on the EPs had results that differed from the individual level (3.2). Graphs displaying relationships between light availability and CWM functional trait values are included in the main manuscript, as these data are presented for the first time and represent a critical component of the study's novel contributions.

**Fig. 6:**
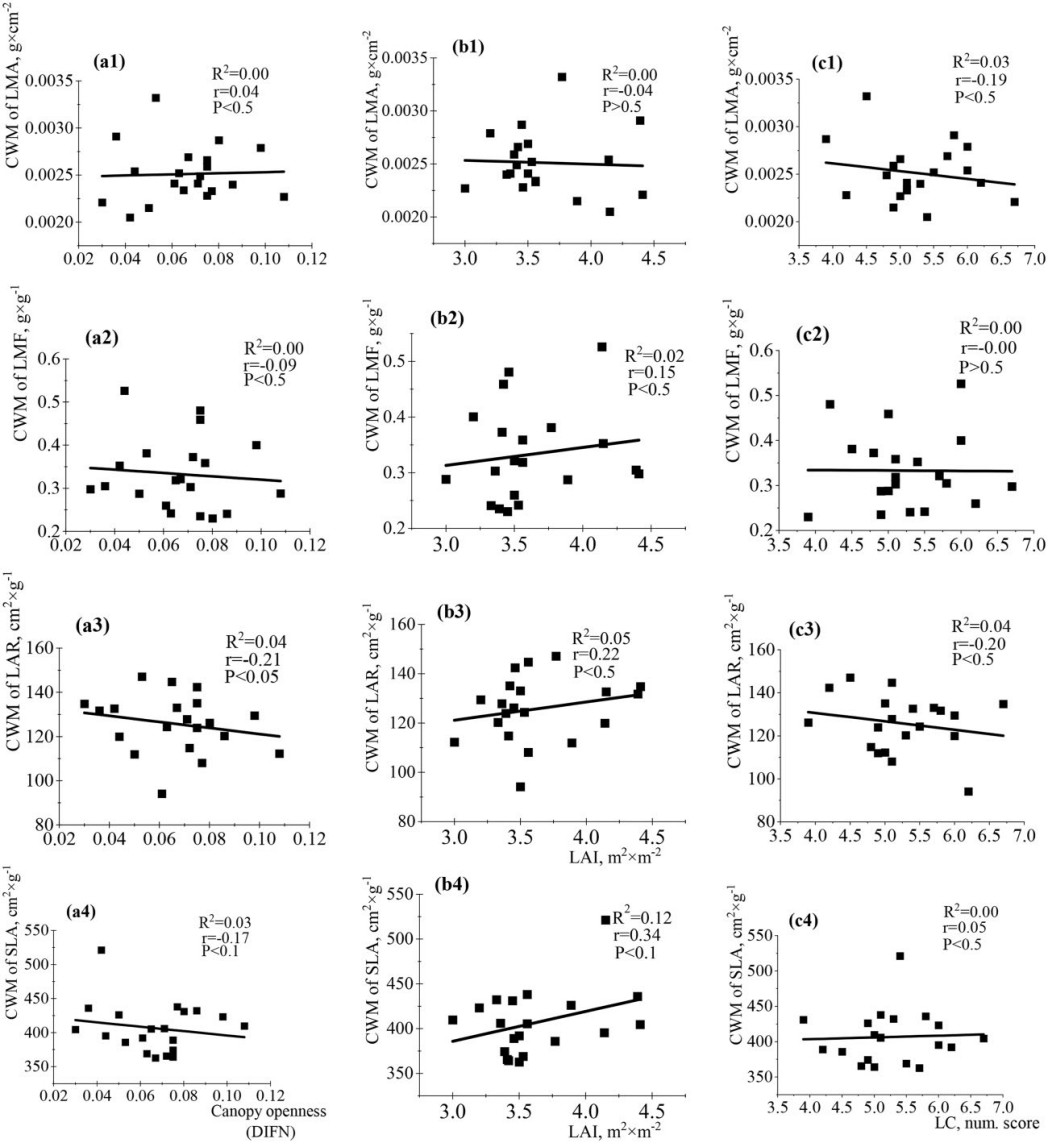
Single-variable linear regression analyses between (A) DIFN (diffuse noninterceptance, representing canopy openness), (B) LAI (leaf area index), (C) LC (light coefficient, derived from Ellenberg values), and the community-weighted mean (CWM) of leaf traits (LMA: leaf mass per area—a1–c1; LMF: leaf mass fraction—a2–c2; LAR: leaf area ratio—a3–c3; SLA: specific leaf area—a4–c4).

**Fig. 7:**
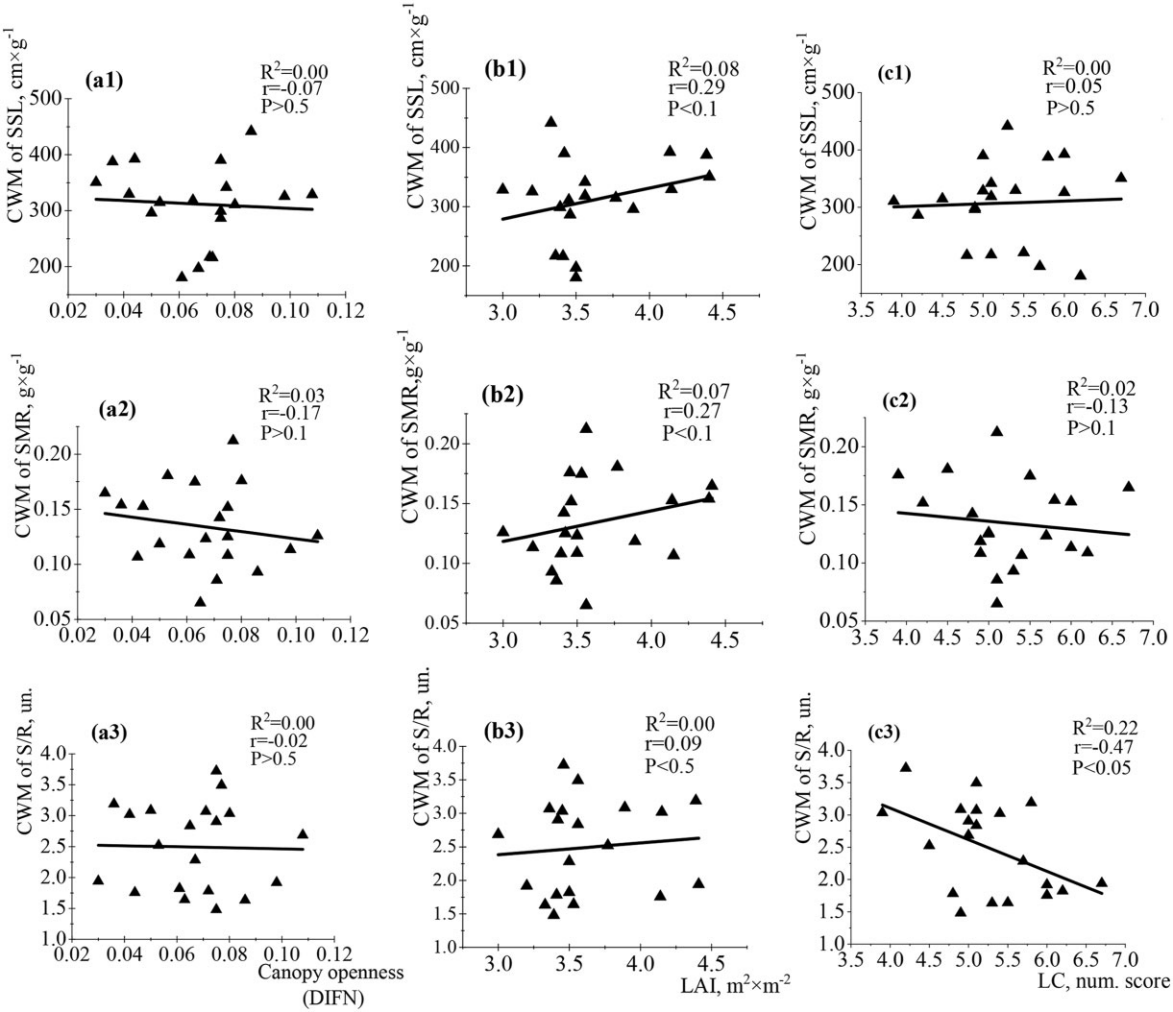
Single-variable linear regression analyses between (A) DIFN (diffuse noninterceptance, representing canopy openness), (B) LAI (leaf area index), (C) LC (light coefficient, derived from Ellenberg values), and the community-weighted mean (CWM) of stem traits (SSL: specific stem length—a1–c1; SMR: stem mass fraction—a2–c2; S/R: stem-to-root ratio—a3–c3).

**Fig. 8:**
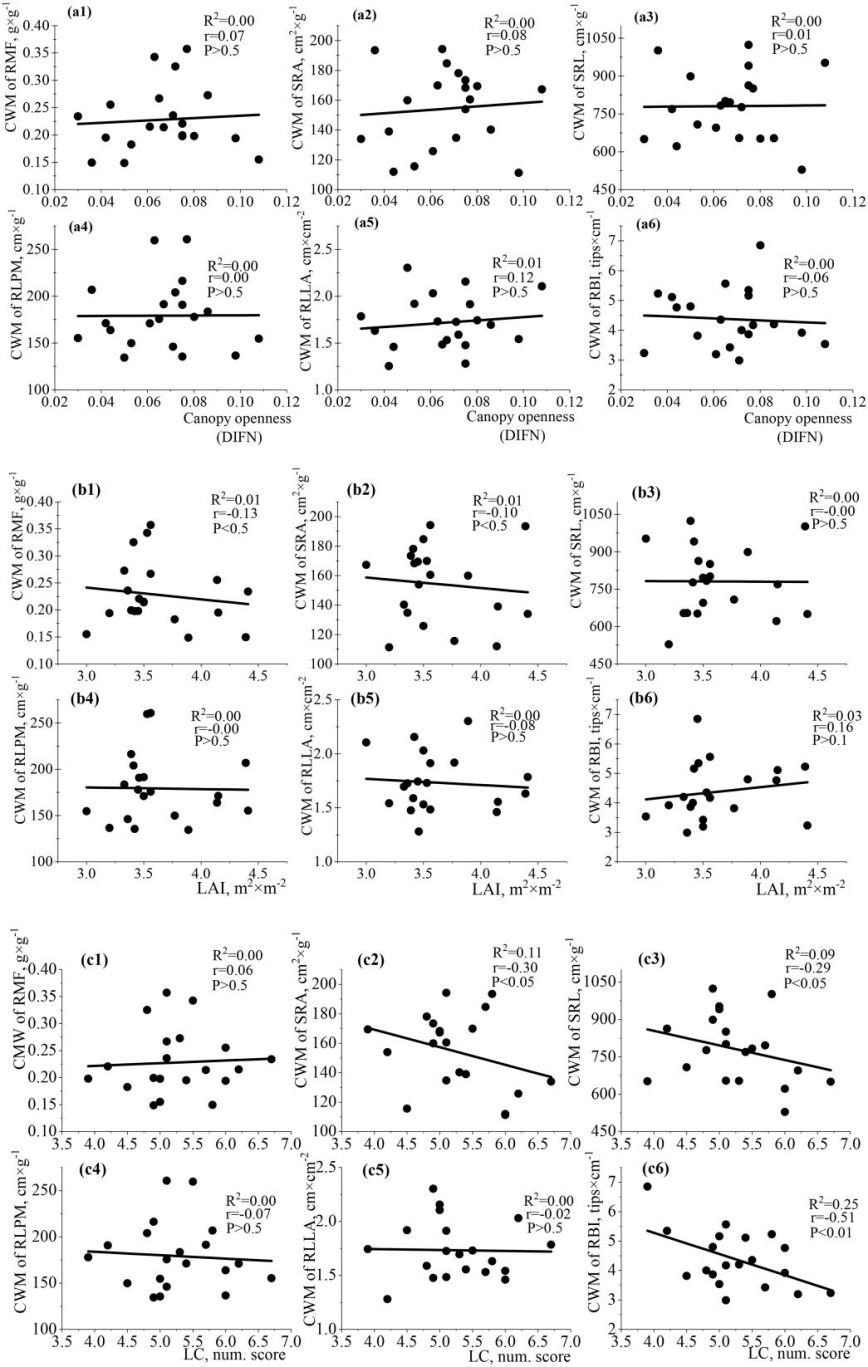
Single-variable linear regression analyses between (A) DIFN (diffuse noninterceptance, representing canopy openness), (B) LAI (leaf area index), (C) LC (light coefficient, derived from Ellenberg values), and the community-weighted mean (CWM) of root traits (RMF: root mass fraction—a1–c1; SRA: specific root area—a2–c2; SRL: specific root length—a3–c3; RLPM: root length per unit plant biomass—a4–c4, RLLA: root length per unit leaf area—a5–c5; RBI: root branching intensity—a6–c6).

The relationships between functional traits of leaves and light changes were not significant at the community-weighed level (Fig. 6). The functional traits of seedling leaves were weakly correlated with DIFN, LAI (direct analysis), and LC (Ellenberg indicator analysis) (Fig. 6). The established relationships were weakly positive or negative. Opposite low correlations between LAI and SLA/LAR; DIFN and SLA/LAR were detected. The correlation between DIFN and SLA (Fig. 6a4) was low (*R*^2^ = 0.03, *r* = −0.17, *P* ˂ 0.1) compared to LAI and SLA (*R*^2^ = 0.12, *r* = 0.34, *P* ˂ 0.1) (Fig. 6b4). The relationships between LAI/LMA and DIFN/LMA had almost the same strength. The LC parameter had negative correlations with LMA and LAR. The relationship between LMA and LC was established only by indirect Ellenberg indicator analysis (*R*^2^ = 0.03, *r* = −0.19, *P* < 0.5, Fig. 6c1). LC was not associated with LMF (*R*^2^ = 0.00, *r* = −0.00, *P* > 0.5) and SLA (*R*^2^ = 0.00, *r* = 0.05, *P* < 0.5) in contrast to LAI and DIFN. The analysis between stem traits and studied parameters of light at the community-weighted level revealed nonsignificant relationships of different strengths and directions (Fig. 7). Positive correlations between SSL and LAI (*R*^2^ = 0.08, *r* = 0.29, *P* ˂ 0.1); SMR and LAI (*R*^2^ = 0.07, *r* = 0.27, *P* ˂ 0.1) were established (Fig. 7b1 and b2). The strength of the relationship between the corresponding stem traits and LAI was closer compared to the corresponding data for DIFN. The relationship between LC and S/R had a significant negative correlation (*R*^2^ = 0.22, *r* = −0.47, *P* < 0.05) (Fig. 7c3), while correlations with LAI and DIFN were not significant (Fig. 7a3 and b3). LC and SSL/SMR relationships were quite weak. Assessment of the relationships between root traits and light availability at the community-weighted level by direct analysis in the Arboretum territory did not detect significant relationships (Fig. 8A and B). The maximum strength of correlations was found for DIFN and RLLA (*R*^2^ = 0.01, *r* = 0.12, *P* ˃ 0.5), LAI and RMF (*R*^2^ = 0.01, *r* = −0.13, *P* ˂ 0.5), and LAI and RBI (*R*^2^ = 0.03, *r* = 0.16, *P* ˃ 0.1). Less close relationships were found for root traits and light parameters compared to leaf/stem traits at this level of analysis. However, negative correlations between LC and RBI (*R*^2^ = 0.25, *r* = −0.51, *P* ˂ 0.01), SRA (*R*^2^ = 0.11, *r* = −0.30, *P* ˂ 0.05) and SRL (*R*^2^ = 0.09, *r* = −0.29, *P* ˂ 0.05) were detected (Fig. 8с6, c2, and c3).

### Light availability and seedling traits

**Fig. 9:**
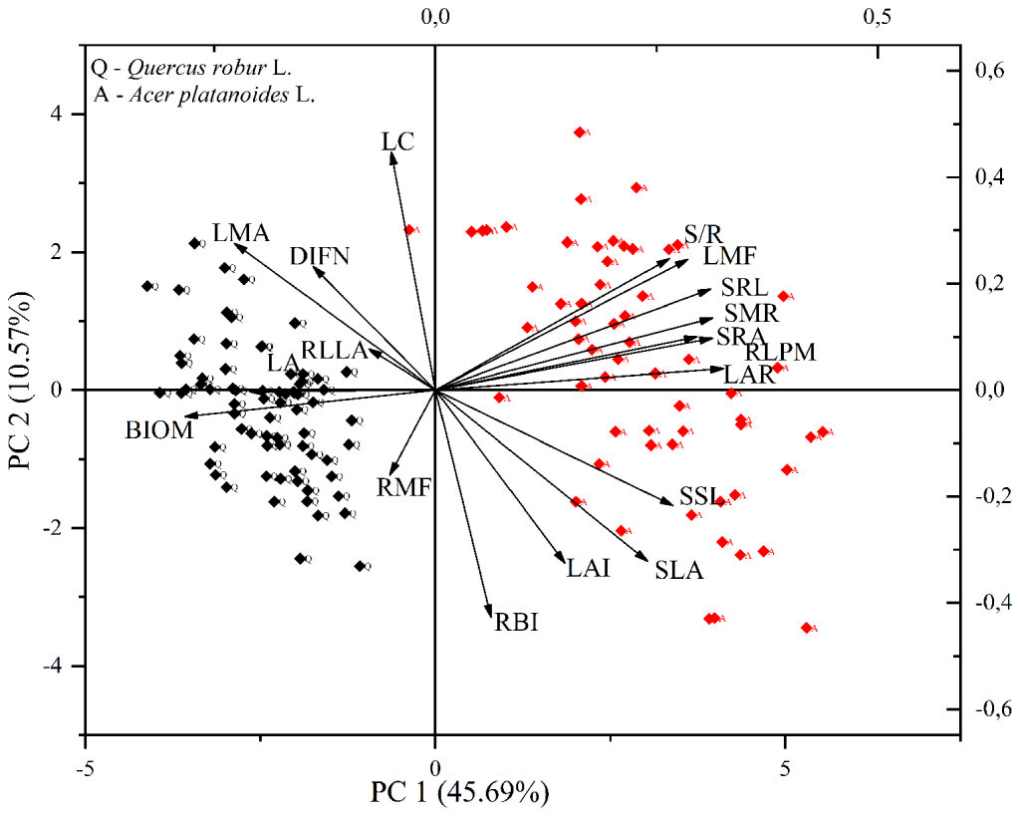
Principal component analysis (PCA) of functional traits of first-year seedlings of *A. platanoides* (A) and *Q. robur* (Q) with light availability (LC: light coefficient, derived from Ellenberg values; LAI: leaf area index; DIFN: diffuse noninterceptance, representing canopy openness). Above-ground traits—SLA: specific leaf area, LMF: leaf mass fraction, LAR: leaf area ratio, LMA: leaf mass per area, SSL: specific stem length, SMR: stem mass fraction, S/R: stem-to-root ratio; and below-ground traits—SRL: specific root length, SRA: specific root area, RMF: root mass fraction, RLPM: root length per unit plant biomass, RLLA: root length per unit leaf area, RBI: root branching intensity.

To understand the multidimensional relationships between the light factors considered in this study, we conducted a PCA. The РСА illustrated the indices of functional trait responses to the light factors by direct and indirect methods (Fig. 9). Vectors for S/R, LMF, SRL, SRA, and SMR had similar directions and lengths and were positively associated with scores on PC1. The main traits affecting PC1 were LAR, RLPM, and SRA in a positive direction, and LA, BIOM, and RLLA in the negative direction. This corresponds to *Acer* (positive) and *Quercus* (negative) for PC1. This clustering is represented by the sites evaluated on *A. platanoides. Quercus robur* showed lower PCA1 scores than *A. platanoides*. RLLA and RMF were the least associated with the two main components according to the PCA. The leaf traits were most associated with the two main components. We can conclude that LAR, LMF, SLA, SRL, and SRA were the most important for PC1. Similarly, we can state that LC and RBI were the most important for PC2.

### Interrelationships of CWM seedling traits

**Fig. 10:**
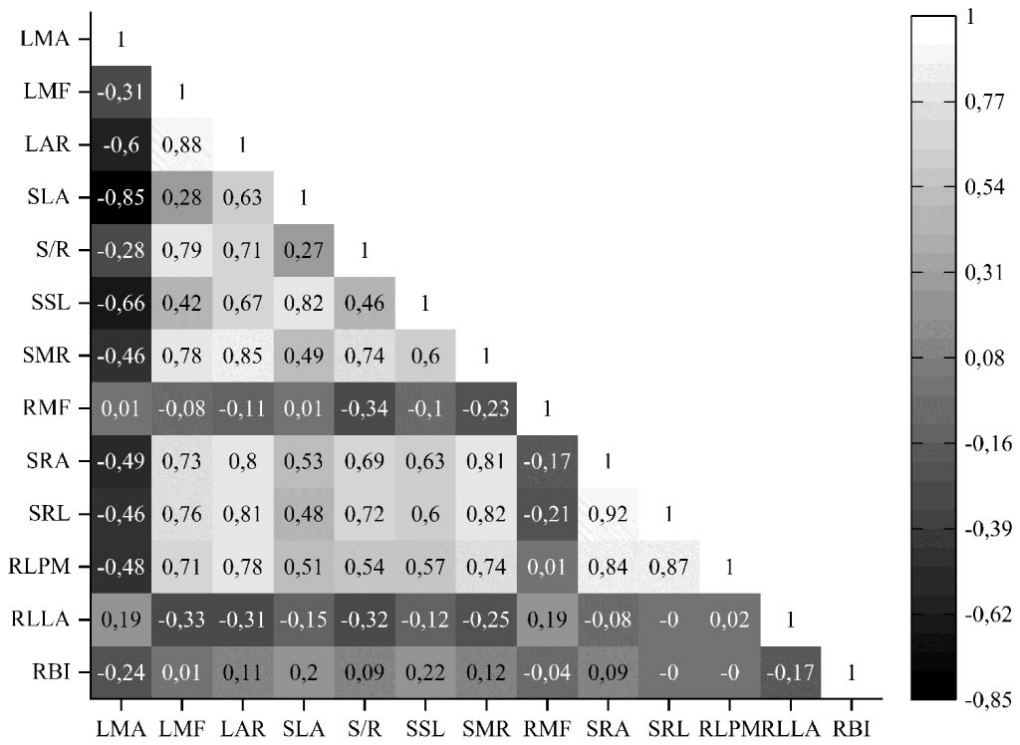
Pearson’s correlations of functional traits for first-year seedlings. Significant (*P* < 0.05) negative or positive correlations between different traits are identified by color (−1.0 to +1.0). Above-ground traits—SLA: specific leaf area, LMF: leaf mass fraction, LAR: leaf area ratio, LMA: leaf mass per area, SSL: specific stem length, SMR: stem mass fraction, S/R: stem-to-root ratio; and below-ground traits—SRL: specific root length, SRA: specific root area, RMF: root mass fraction, RLPM: root length per unit plant biomass, RLLA: root length per unit leaf area, RBI: root branching intensity.

An assessment of correlations between the CWM functional traits of seedlings was carried out in the studied EPs (Fig. 10). Correlations reflecting relationships within the same organ system (e.g., between leaf traits) aligned with ecological expectations. In contrast, correlations representing relationships across different organ systems (e.g., between leaf and root traits) revealed unexpected patterns that warrant further exploration. In particular, strong positive correlations between LMF and RLPM (0.71), LMF and SRL (0.76), and LMF and SRA (0.73) were established (Fig. 10). Very strong relationships between LAR and RLPM (0.78), LAR and SRL (0.81), and LAR and SRA (0.80) were also detected. The highest correlations among stem/leaf traits relationships were found between LAR and SMR (0.85), LMF and SMR (0.78), and SLA and SSL (0.82). It should be noted that SLA was not closely related to other stem and root traits. The relationships between SSL and LMA/LAR were similar in strength and opposite in direction. Strong positive correlations were also found between SMR and RLPM/SRL/SRA (0.74, 0.82, and 0.81, respectively).

## Discussion

This study provides novel insights into the relationships between natural light availability and functional traits of *A. platanoides* (shade-tolerant) and *Q. robur* (shade-intolerant) seedlings, assessed using both direct (LAI, DIFN) and indirect (Ellenberg indicator values) methods. The study integrates individual- and community-level trait analyses to evaluate functional traits of these species along natural light gradients, revealing their distinct adaptive strategies. Key findings include (1) stronger correlations between individual-level trait data and light availability compared to community-level data; (2) confirmation of the variation in phenotypic plasticity of functional traits in response to local light conditions, highlighting the adaptive responses of seedlings to heterogeneous light environments; and (3) detection of the closest relationships between functional traits within the same organ system and links between traits of different organ systems in *A. platanoides* and *Q. robur* seedlings, providing further insights into their adaptive strategies under varied environmental conditions.

The contrasting phases of tree growth from seedlings to mature plants and different plant organs (i.e., leaf, stem, and roots) are priority areas for the study of forest ecology (He et al. 2019, 2020). Interspecific variation in demographic rates is more important for small plants than for large trees, which makes it statistically easier to detect trait–abiotic factor relationships. Seedling functional type is a categorical trait that can be used to characterize plant regenerative strategies and plant adaptation to environmental changes (Perez-Harguindeguy et al. 2013). Most of the studies have been carried out on first-year tree seedlings with phanerocotylar epigeal germination with foliaceous cotyledons and cryptocotylar hypogeal germination with reserve storage cotyledons (Khan et al. 2021). Traits of these seedling functional types influence the growth, reproduction, and survival of seedlings in the future (Lavorel and Garnier 2002; Violle et al. 2007; He et al. 2020) and are very important indices for understanding how seedlings respond and adapt to changing light conditions (Perez-Harguindeguy et al. 2013; He et al. 2019). The different directions and strengths of the relationship patterns of functional traits were characterized by previous studies (Fortunel et al. 2012). Traits of leaf, stem, and root delineated two orthogonal axes of functional trade-offs: a first axis defined by leaf traits, corresponding to a “leaf economics spectrum,” and a second axis defined by covarying stem and woody root traits, corresponding to a “wood economics spectrum.” These axes remained consistent when accounting for species evolutionary history with phylogenetically independent contrasts (Fortunel et al. 2012).

Light is a limiting factor for seedlings (Dyderski and Jagodziński 2018). The seedling morphological response to the availability of light could be a major key that differentiates ecological niches of species. Previous studies have shown that seedling spatial distribution is significantly associated with light. For instance, Xia et al. (2019) reported that light heterogeneity promoted seedling growth but had no discernible effects on seedling survival. Previous results showed that seedlings of temperate and tropical plants are limited by light in shaded habitats (Uriarte et al. 2005; Han et al. 2017), and variation in functional traits of trees is expected to help capture the variation in light (Reich et al. 1998; Poorter and Rose 2005; Coble and Cavaleri 2014). The plant economics spectrum in the seedling community suggests that limitation by canopy openness, often used as a proxy for light availability, is not the major factor driving the functional response in forest ecosystems (Shen et al. 2019). However, this conclusion is specific to their study context and does not necessarily exclude the potential role of direct light intensity measurements in explaining functional responses in other ecological settings. The survival rates of shade-tolerant species will not be as strongly influenced by light. Shade-intolerant species, however, do not tolerate low light levels (Han et al. 2017). Seedlings of the different species differentially allocate biomass to stems, leaves, and roots in response to differences in light levels (Saldaña-Acosta et al. 2009). Plant biomass increased and shoot–root ratio decreased with light availability. The interaction between species and natural light gradients was significant when plant biomass was taken as a covariate, revealing significant species differences in their plastic phenotypic response to light that were not simply due to differences in plant size (Valladares et al. 2000).

The results obtained in this study indicate a slight variation in the values of biomass, as well as the leaf/root functional traits of shade-tolerant *A. platanoides* and shade-intolerant *Q. robur* seedlings along a natural gradient of light availability. These results reflect naturally occurring differences in light conditions, which were not experimentally manipulated but quantified using direct and indirect methods to capture the ecological relevance of seedling responses in situ. Correlations between seedling above-ground biomass and morphological traits are strongest at low irradiance, where light interception is important (Umana et el. 2021). Conversely, correlations between seedling mass and physiological traits are strongest at high irradiance, where maximization of photosynthetic rates is important (Poorter and Rose 2005). The correlated links between studied leaf traits of shade-tolerant and shade-intolerant species and light availabilities at the individual level of analysis were shown in this study. The relationships between studied root/stem traits of both species and light availabilities at the individual level of analysis were weaker in comparison to leaf traits. The obtained negative and positive relationships between various functional traits of first-year maple and oak seedlings were related to the stability of the development of individuals in the studied abiotic conditions. We did not measure temporal heterogeneity of light since we only measured canopy openness in one growing season (2023). Therefore, we agree with authors (Shen et al. 2019) that unidentified temporal variation in canopy openness could also contribute to the link between seedling traits and the light environment.

Shade-intolerant species were characterized by a relatively small proportion of their saplings surviving or establishing in low-light conditions, fast height growth, and a strong growth response to light (Poorter and Arets 2003; Poorter et al. 2012). The first-year seedlings of shade-intolerant species have high population-level mortality rates in the shaded understory (Wright et al. 2004). The seedlings of shade-intolerant species responded to the shading with reduced above-ground biomass. The shade-intolerant species (*Q. robur*) exhibited reduced survival in low-light conditions due to lower allocation to below-ground biomass, reflecting its ecological strategy of prioritizing rapid above-ground growth to capture light in high-light environments. These findings are consistent with previous research ( Kozlowski and Pallardy 2002) and emphasize the trade-offs inherent in shade-intolerant strategies.

Conversely, the shade-tolerant species (*A. platanoides*) maintained more balanced biomass allocation, particularly through higher investments in root traits (e.g., RMF, SRL), which support survival in shaded conditions. This aligns with prior studies (Poorter et al. 2012) while also highlighting the higher phenotypic plasticity observed in leaf traits of shade-tolerant species. These findings provide new evidence that phenotypic plasticity in traits like SLA and LAR contributes significantly to the ecological strategies of shade-tolerant (*A. platanoides*) species under natural light gradients.

Seedlings put more emphasis on shoot growth than root growth, which is a common response of tree seedlings to variation of light intensity (morphological adaptations) (Kozlowski and Pallardy 2002). Studies with seedlings invariably show that SLA is an important predictor of interspecific variation in growth and survival (Kitajima 1994; Poorter and Rosc 2005; Poorter et al. 2012). Leaves of those seedlings growing in poorly lit conditions had a higher SLA and a higher biomass allocation to stems and leaves relative to root allocation compared to those growing in less limited light conditions (Saldaña-Acosta et al. 2009). Some studies have not observed strong correlations between leaf/root economic traits and showed higher complexity of environmental constraints and plant functions below ground than above ground (Weemstra et al. 2016). Our results are consistent with studies that have found coordination between leaves and roots (Freschet et al. 2010; Perez-Ramos et al. 2012).

SRL has been viewed as a below-ground analogue to SLA, where high SRL might facilitate faster growth through more rapid acquisition of soil resources (Kramer-Walter et al. 2016). Substantial trait coordination occurred between leaves and roots, but the strength varied between growth forms and clades (Valverde-Barrantes et al. 2017). The slope between LMF and RMF shows no relationship with irradiance, whereas the slope of SMR versus seed mass increases with irradiance (Poorter and Rose 2005). Higher allocation to roots can occur early in ontogeny (Lohier et al. 2014). The plasticity of S/R ratios can be a function of different environmental factors (Bonifas et al. 2009) or even ecotypes with different strategies of resource uptake (Maškova and Herben 2018). A statistical relationship between S/R and light parameters measured by direct methods was not detected in this study, in contrast to the relationship with data obtained by indirect methods. Seedlings increased horizontal growth relative to vertical growth in environments with greater irradiance. Root mass ratio increased with increasing irradiance in shade-intolerant and shade-tolerant species and was not different between the species (Niinemets 1998; Niinemets and Valladares 2006). Across the larger environmental gradient, leaf and root functional traits were significantly correlated (Wright et al. 2004). Positive correlations between leaf traits and root traits were also established in this study (LMF and RLPM, LMF and SRL, LMF and SRA, etc.). This conclusion indicates an intrinsic trade-off between the resource allocation into more efficient absorbing surfaces of either leaves or roots (Wright et al. 2007). This study confirms the hypothesis that root traits are more than analogues of leaf traits within a plant economics spectrum. The results reveal a novel ecological pattern and highlight the power of root data to close important knowledge gaps in trait-based plant ecology (Bergmann et al. 2017).

Shade-tolerant *A. platanoides* seedlings had higher densities, recruitment, and survival than shade-intolerant *Q. robur* (Reinhart et al. 2006). *Acer platanoides* exhibits a phanerocotylar epigeal functional type with foliaceous cotyledons, emphasizing its adaptation to optimize light capture in shaded understory conditions. This strategy enables early photosynthetic activity, enhancing carbon gain in low-light environments (Kitajima 1994). Conversely, *Q. robur* demonstrates a cryptocotylar hypogeal functional type with reserve storage cotyledons, prioritizing rapid root development and resource storage (Khan and Gul 2006). This adaptation supports establishment in competitive environments by securing below-ground resources and withstanding resource-limited conditions. These functional types highlight the ecological divergence and adaptive strategies of the two species during early development.

Biomass allocation between different compartments was also dependent on seedling size. With increasing canopy openness (quantified as LAI and DIFN), shade-tolerant *A. platanoides* maintained a constant proportion of biomass in foliage, but the relative amount of foliage decreased in shade-intolerant *Q. robu*r. As a result of these interspecific differences in fractional allocation of sapling biomass in foliage and biomass requirement for construction of foliar surface area, LAR was larger in *A. platanoides. Quercus robur* had a larger SMR (van Hees 1997; Welander and Ottosson 1998) and SLA in the lowest light levels (Saldaña-Acosta et al. 2009), while *A. platanoides* had a greater LMR/LMA (Niinemets 1998; Gomez-Aparicio et al. 2008). Although shade treatments significantly affected oak seedling growth, they did not influence seedling survival. The biomass increased as light intensity increased. Oak seedlings showed typical acclimation to shade (Welander and Ottosson 1998), including greater SLA and lower leaf thickness and root: shoot ratios with increasing shade (Valladares et al. 2002). The results of another study suggested that *Q. robur* seedlings would have high survival rates and would acclimate well if underplanted below overstories that reduced the available light to as low as 28% of full light (Sevillano et al. 2016). Positive correlations between LAI and SLA and LAI and LAR of first-year oak seedlings were detected in our study. The data obtained in this study regarding the response of functional traits of shade-tolerant *A. platanoides* to light availability are consistent with the results of other authors. Shade-tolerant *A. platanoides* responds to even small increments in light intensity because its foliage characteristics allow more efficient light capture (Lapointe and Brisson 2012). We confirmed that slightly increased light caused a change in the values of morphological parameters of leaf traits of shade-tolerant *A. platanoides*. We agree with the conclusion that due to a decreasing investment of resources in foliage construction with advancing seedling ontogeny, first-year seedlings require more light to survive in *Q. robur* than in *A. platanoides* (Lapointe and Brisson 2012).

These results indicate that interspecific differences significantly alter species competitive relationships during seedling development in *ex situ*. These species differences in their functional response to light reinforce the view of a different regeneration niche for each of the two species studied. The different response of functional traits of both species to light changes could stem from the fact that shade-intolerant species with traits adapted for fast resource acquisition (e.g., high LAR, SSL, and SRA) were more abundant in the seedling community (Record et al. 2016). Greater investment of biomass in leaves vs. standing biomass may result in lower volume gain and reduced competitive ability in more open habitats in shade-tolerant *A. platanoides*. This is in contrast to shade-intolerant *Q. robur*, which not only tolerates full sunlight but also requires increased irradiance after the first year of emergence for successful regeneration (Welander and Ottosson 1998).

Ecological assessment based on only a specific biomass component is potentially misleading because plasticity differs across the complete dose–response continuum and is further driven by a series of factors. Seedling trait estimates should be based on several components (Agathokleous et al. 2019). A multifaceted approach was also used in our study. Plasticity occurs in the context of optimal partitioning theory; however, it is constrained by plant ontogeny (Lohier et al. 2014). Trait plasticity is an important factor affecting upscaling from individual to community-level biomass productivity. The establishment of the function–performance relationships that should lead to more robust predictions is a debatable issue. Our study of phenotypic plasticity prioritized species-level comparisons by employing mean trait values across environmental gradients to calculate the phenotypic PI. This approach, grounded in established methodologies (Valladares et al. 2000), offers a reliable framework for interspecific comparisons. However, it inherently overlooks within-species variability, potentially leading to an underestimation of the full extent of phenotypic flexibility at the individual level. Notably, the significant variability observed in traits such as SLA and RLLA highlights the need for future investigations to incorporate individual-level calculations of phenotypic PI. Such an approach would enable a deeper exploration of phenotypic responses to environmental heterogeneity, especially in contexts where light availability exhibits considerable spatial variation.

The PIs in this study were calculated using mean values to provide a generalized representation of species-level responses. This approach ensures comparability between the two species and minimizes noise from excessive individual variability. The selected approach (Valladares et al. 2000) allows for the assessment of plasticity at the species level and is widely accepted for interspecific comparisons in natural conditions. Furthermore, other studies confirm the appropriateness of this method for evaluating plasticity in natural environments, which reinforces the validity of our results (Cadena-Zamudio et al. 2024). However, we recognize that this method does not fully capture within-population variation. To address this, we calculated coefficients of variation (CV), which demonstrated substantial variability in functional traits for both species (e.g., stem traits: 29.16–69.18%, root traits: 30.34–67.45%). In future research, calculating PIs for each individual and then averaging these values could further enhance the robustness of our results and provide additional insights into within-population dynamics. The issue of the value of phenotypic PIs of first-year seedlings of species in different environmental conditions (e.g., light) is still questionable.

The shade-intolerant species showed higher values of photosynthetic PIs than shade-tolerant species, according to some studies (Strauss-Debenedetti and Bazzaz 1996; Valladares and Niinemets 2008). The results of other studies suggest that mean phenotypic plasticity was similar for shade-tolerant and shade-intolerant species (van Hees 1997; Valladares et al. 2002, 2006, 2015). Variation in light availability was clearly related to variation in sapling growth (Finzi and Canham 2000).

Second-year seedlings of shade-intolerant *Q. robur* were more plastic in physiological features (Valladares et al. 2002, 2006, Valladares and Niinemets 2008) in contrast to first-year seedlings (van Hees 1997). Resources of the acorn can significantly impact the productivity of first-year seedlings (Welander and Ottosson 1998). Phenotypic plasticity for morphological traits was not high in shade-intolerant *Q. robur*. Maximum photosynthetic rates increased with increasing light availability in shade-intolerant *Q. robur*. These results suggest that a greater tolerance of strong irradiance is linked to enhanced physiological plasticity of variables related to photosynthesis, while shade tolerance relies on enhanced plasticity of light-harvesting variables (morphology) (Valladares et al. 2002, 2006).

This study found that the phenotypic PIs for all variables rendered a similar value for the two species. Phenotypic plasticity can increase both community-wide trait means or trait variations, i.e., increase effects at the community level both via the biomass ratio or the diversity hypothesis (Luo et al. 2020). Other studies, focused on the diversity hypothesis, also found that trait plasticity in response to species richness typically increased community productivity (Sapijanskas et al. 2014). Native species showed similar levels of resource-use efficiency and there was no relationship between species plasticity and resource-use efficiency across species (Funk 2008). This was confirmed in our study. However, some studies only analyzed community-level effects, and thus did not test how individual-level effects were related to these. CWM represents the community structure of each seedling plot and allows a link to their environmental conditions (de la Riva et al. 2016a, 2016b; Johnson et al. 2017). This was confirmed in our results, where relationships between measures obtained from individual-level trait data were stronger than relationships with measures obtained from community-level trait data. We showed that the CWM seedling traits were less correlated with canopy openness and LAI. Our results are consistent with previously reported studies. The plant traits showed significant correlations with canopy openness; LAR, SSL, and SRA were positively connected to canopy openness, while LA and RTD were negatively correlated (Shen et al. 2019). The maximum entropy model has been shown to explain significant variation in species abundances based on their functional traits (Sonnier et al. 2010; Merow et al. 2011; Shipley et al. 2011). Our results are consistent with studies that propose that survivorship is driven by interspecific differences in resource uptake and tolerance (Canham et al. 1994).

The differences in the data obtained by different analysis methods, from our point of view, were related to the locality of the conducted study and the range of investigated ecological conditions that occurred in the Arboretum. Uncovering the links between limiting abiotic factors, functional trait diversity, and performance is necessary to better predict seedling responses to changes in abiotic conditions in the future.

Future research should focus on addressing several critical areas to advance the understanding of seedling functional traits and their ecological implications. First, investigating temporal heterogeneity in light availability across multiple growing seasons is essential to capture the dynamic fluctuations in canopy openness and their influence on seedling traits. Second, incorporating individual-level measurements to calculate phenotypic PIs is necessary to account for within-population variability. Such detailed analyses would enhance the precision of trait-based studies and reveal finer-scale patterns of plastic responses to environmental gradients. Lastly, future studies should explore a broader range of abiotic factors, including soil moisture and nutrient availability, to understand their interactive effects on phenotypic plasticity.

## Conclusion

The morphological and ecological characteristics of seedlings are critical aspects in understanding the adaptation of trees at this ontogenetic phase to environmental changes. In this study, we tried to determine the patterns of response of functional traits of a shade-tolerant (*A. platanoides*) and a shade-intolerant (*Q. robur*) species along natural environmental light gradients, using an entire-level, trait-based approach by direct and indirect methods. The obtained data on the values of functional traits of *A. platanoides* and *Q. robur* indicated that relationships between measures obtained from individual-level trait data were stronger than relationships with measures obtained from community-level trait data in the studied environmental conditions. The variation in the phenotypic plasticity of functional parameters of seedlings and local light levels was confirmed. Relationships between traits of different organ systems and traits within the same organ system parameters of the functional traits of *A. platanoides* and *Q. robur* seedlings were detected. Our results are required for successful resumption of maple and oak in *ex situ* conservation. We suggest that this type of study be conducted for human-impacted forests and parks.

## Author contributions

O.B.: conceptualization, data curation, formal analysis, funding acquisition, investigation, methodology, software, supervision, validation, visualization, writing—original draft, writing—review & editing. R.J.: conceptualization, data curation, formal analysis, investigation, methodology, project administration, resources, software, validation, visualization, writing—review & editing. A.M.J.: conceptualization, funding acquisition, investigation, methodology, project administration, supervision, validation, writing – original draft, writing – review & editing.

## Supplementary Material

icaf003_Supplemental_File

## Data Availability

The original contributions presented in the study are included in the article/supplementary material. Further inquiries can be directed to the corresponding author.
