# Supplementary material for: The Impact of Light Availability on the Functional Traits of Quercus Robur L. and Acer Platanoides L. First-Year Seedlings by Direct and Indirect Methods
Source: Integr Comp Biol. 2025 Jan 30;65(6):1312–35. doi: 10.1093/icb/icaf003 (PMC12690478; doi:10.1093/icb/icaf003)
Supplement: icaf003_Supplemental_File [file icaf003_supplemental_file.docx]

**Supplementary data**

**Table S1**. The definitions, abbreviations, and units for studied functional traits of individual seedlings

| Above-  ground traits | **Trait** | **Abbr.** | **Definition** | **Unit** |
| --- | --- | --- | --- | --- |
|  | Total seedling biomass | BIOM | Seedling dry entire biomass | g |
|  | Leaf area | LA | Leaf surface area | cm^2^ |
|  | Leaf mass per area | LMA | The leaf dry biomass per unit leaf area | g×cm^-2^ |
|  | Leaf mass fraction | LMF | The ratio of leaf dry biomass to the dry biomass of the entire individual | g×g^-1^ |
|  | Leaf area ratio | LAR | The ratio of total leaf area to dry entire biomass | cm^2^×g^-1^ |
|  | Specific leaf area | SLA | The ratio of leaf area to leaf dry biomass | cm^2^×g^-1^ |
|  | Specific stem length | SSL | The ratio of stem length to stem dry biomass | cm×g^-1^ |
|  | Stem mass fraction | SMR | The ratio of stem dry biomass to dry entire biomass | g×g^-1^ |
|  | Stem root ratio | S/R ratio | The ratio of stem dry biomass to dry entire biomass | g/g |
| Below-  ground traits | Root mass fraction | RMF | The ratio of root dry biomass area to dry entire biomass | g×g^-1^ |
|  | Specific root area | SRA | The ratio of total root area to dry entire biomass | cm^2^×g^-1^ |
|  | Specific root length | SRL | The ratio of root length to root dry biomass | cm×g^-1^ |
|  | Root length per unit plant biomass | RLPM | The ratio of root length to dry entire biomass | cm×g^-1^ |
|  | Root length per unit leaf area | RLLA | The ratio of root length to total leaf area | cm×cm^-2^ |
|  | Root branching intensity | RBI | The ratio of the number of root tips to length of first order root | tips×cm^-1^ |

**Figure S1.** Single-variable linear regression analyses between LAI (leaf area index) and below-ground traits (SRA: specific root area – a, SRL: specific root length – b, RLLA: root length per unit leaf area – c) of *A. platanoides* seedlings. Each point represents individual seedling measurements.


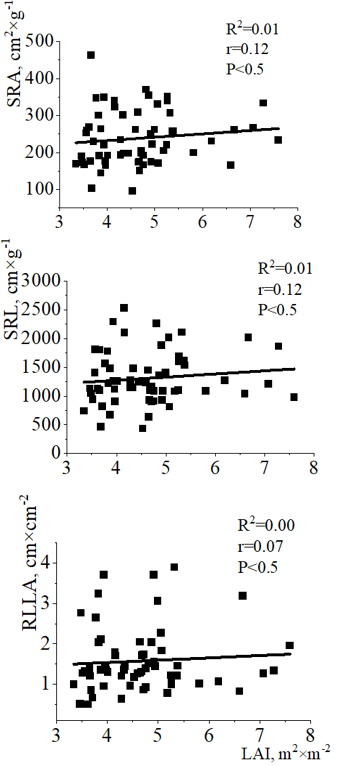


**(c)**

**(b)**

**(a)**

**Figure S2.** Single-variable linear regression analyses between LAI (leaf area index) and above-ground traits (S/R: stem-to-root ratio – a, SMR: stem mass fraction – b) and below-ground traits (RMF: root mass fraction – c, SRA: specific root area – d, RLPM: root length per unit plant biomass – e, RLLA: root length per unit leaf area – f, RBI: root branching intensity – g) of *Q. robur* seedlings. Each point represents individual seedling measurements.


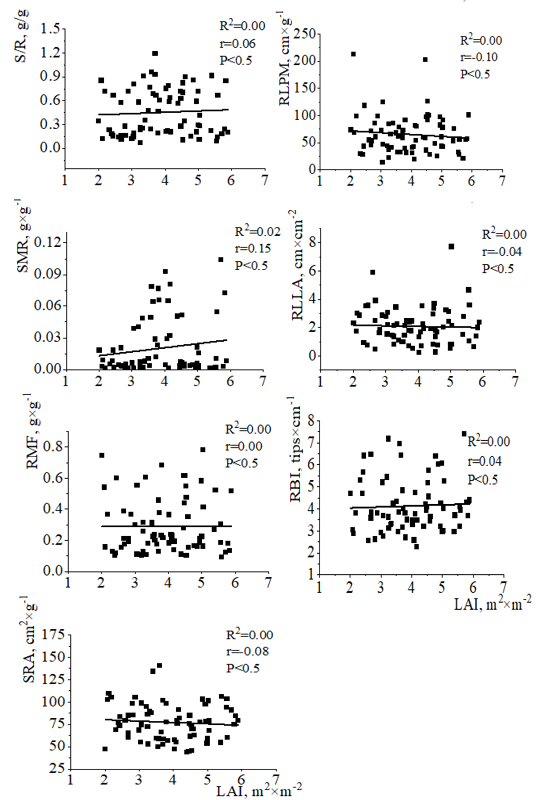


**(g)**

**(f)**

**(e)**

**(d)**

**(c)**

**(b)**

**(a)**
